# Soil pathogen communities associated with native and non-native *Phragmites australis* populations in freshwater wetlands

**DOI:** 10.1002/ece3.900

**Published:** 2013-12-03

**Authors:** Eric B Nelson, Mary Ann Karp

**Affiliations:** Department of Plant Pathology and Plant-Microbe Biology, Cornell University334 Plant Science Building, Ithaca, New York, 14853-4203

**Keywords:** Oomycetes, plant invasions, plant–soil feedbacks.

## Abstract

Soil pathogens are believed to be major contributors to negative plant–soil feedbacks that regulate plant community dynamics and plant invasions. While the theoretical basis for pathogen regulation of plant communities is well established within the plant–soil feedback framework, direct experimental evidence for pathogen community responses to plants has been limited, often relying largely on indirect evidence based on above-ground plant responses. As a result, specific soil pathogen responses accompanying above-ground plant community dynamics are largely unknown. Here, we examine the oomycete pathogens in soils conditioned by established populations of native noninvasive and non-native invasive haplotypes of *Phragmites australis* (European common reed). Our aim was to assess whether populations of invasive plants harbor unique communities of pathogens that differ from those associated with noninvasive populations and whether the distribution of taxa within these communities may help to explain invasive success. We compared the composition and abundance of pathogenic and saprobic oomycete species over a 2-year period. Despite a diversity of oomycete taxa detected in soils from both native and non-native populations, pathogen communities from both invaded and noninvaded soils were dominated by species of *Pythium*. Pathogen species that contributed the most to the differences observed between invaded and noninvaded soils were distributed between invaded and noninvaded soils. However, the specific taxa in invaded soils responsible for community differences were distinct from those in noninvaded soils that contributed to community differences. Our results indicate that, despite the phylogenetic relatedness of native and non-native *P. australis* haplotypes, pathogen communities associated with the dominant non-native haplotype are distinct from those of the rare native haplotype. Pathogen taxa that dominate either noninvaded or invaded soils suggest different potential mechanisms of invasion facilitation. These findings are consistent with the hypothesis that non-native plant species that dominate landscapes may “cultivate” a different soil pathogen community to their rhizosphere than those of rarer native species.

## Introduction

Interactions of introduced plants with soil microbial communities are increasingly viewed as important determinants of invasive success (van der Putten et al. 2007; Inderjit and van der Putten 2010; Mordecai 2011; Steinlein 2013). However, specific microbial communities that may facilitate invasions have rarely been characterized, and their interactions with native and non-native plant species are poorly understood. The concept of plant–soil feedback (PSF) has become a widely accepted framework for understanding how interactions of plants with soil microbes may influence plant community dynamics (Bever et al. 2012) and plant invasions (Inderjit and van der Putten 2010; Steinlein 2013). The ecological foundations of this theory predict that plants, by releasing exudates and rhizodeposition products from belowground organs, influence growth and activity of soil microbes in a plant-specific manner (Bever et al. 2012). This enhanced microbial activity then feeds back to influence plant growth and competitiveness in either a positive or negative way (Perkins and Nowak 2012).

The direction and strength of plant responses to soil microbes represent the net result of both positive and negative PSFs. Positive PSFs may arise from the accumulation of growth-promoting mutualists, such as mycorrhizal fungi, nitrogen-fixing bacteria, or other root-associated microbes (Pringle et al. 2009; Shah et al. 2009), whereas negative PSFs are thought to result from plant-specific increases in abundance and/or virulence of soil pathogens and herbivores (Bever 2003). While positive PSFs may facilitate dominance of some highly competitive species, negative PSFs serve to limit the dominance of any one species while promoting the coexistence of diverse species (van der Heijden et al. 2008).

A large body of evidence now indicates that many of the observed negative PSFs are driven by soil pathogens (Klironomos 2002; Kardol et al. 2007; Maron et al. 2011; Schnitzer et al. 2011; de Kroon et al. 2012). Yet, much of the empirical evidence for soil pathogen-mediated negative PSFs is indirect with no direct experimental evidence for the changes in soil pathogen communities that accompany changes in plant performance. Inferences linking plant performance with selective soil microbial enhancement commonly rely solely on comparative plant growth responses in soils conditioned either by the introduced non-native or the native species. In some cases, conditioned soils are either treated with fungicides or sterilized (Reinhart et al. 2003, 2005; Callaway et al. 2004; Nijjer et al. 2007; MacKay and Kotanen 2008; Petermann et al. 2008; Reinhart and Clay 2009; Flory et al. 2011; Maron et al. 2011; Orrock et al. 2012; Phillips and Murray 2012). Because these treatments eliminate large portions of the microbial diversity present in soils, including both pathogens and nonpathogens, the interpretation of the results from such treatments is particularly challenging when attempting to ascribe cause and effect. Without any knowledge of the pathogen and other microbial community changes that accompany soil conditioning by invasive plant species, actual mechanisms underlying PSF responses and their role in invasive success remain obscure.

Within the PSF framework, soil pathogen communities are predicted to respond to plants in several ways. If soil pathogens limit the productivity of native noninvasive plants, then non-native species that become dominant in the landscape should either (1) accumulate fewer or less virulent pathogens relative to rarer native species (Klironomos 2002) or (2) differentially amplify pathogens that potentially spillback onto native noninvasive species (Eppinga et al. 2006; Nijjer et al. 2007; Mangla et al. 2008) rendering them less competitive. Additionally, although introduced non-native plant species may initially have higher levels of resistance or tolerance to pathogens leading to a local release from pathogen impacts, this is likely to be temporary (Hawkes 2007; Diez et al. 2010; Phillips et al. 2010; Flory et al. 2011). Regardless, of the actual mechanism by which soil pathogens respond to soil conditioning by the introduced non-native plant species, the negative impacts on plant performance should be greater on the native species than on the non-native species and should be reflected either in differences in the spectrum of pathogenic taxa or in the relative abundance and/or virulence of specific soil pathogens present in soils of native and non-native species.

Increasing evidence implicates oomycete plant pathogens as important contributors to negative PSFs (e.g., Westover and Bever 2001; Packer and Clay 2004; Augspurger and Wilkinson 2007; Callaway et al. 2011; Reinhart et al. 2011; Gomez-Aparicio et al. 2012). Oomycetes [supergroup Chromalveolata, kingdom Chromista (Beakes et al. 2012)] are among the most diverse and widespread group of pathogens found in terrestrial and aquatic habitats, with hosts ranging from plants, invertebrates, and vertebrates to fungi, algae, and other microbes (Dick 2001). Despite broad pathogenic lifestyles, oomycetes are most notorious for their widespread pathogenicity to plants (Levesque 2011). Aside from a limited number of studies implicating oomycete pathogens as potentially important in negative PSFs and plant invasions (Klironomos 2002; Zhang et al. 2009; Reinhart et al. 2010, 2011), few studies have examined communities of oomycete pathogens in natural ecosystems (Nechwatal et al. 2008a; Wielgoss et al. 2009).

In the present study, we examine the impacts of native and non-native populations of *Phragmites australis* on communities of soil oomycete pathogens. We focus on *Phragmites australis* (European common reed) not only because of its importance as an invasive plant species in North American wetlands (Plut et al. 2011), but also because of the many native noninvasive haplotypes sympatric with one non-native *P. australis* haplotype (haplotype M) of European origin (hereafter referred to as *australis*) in invaded wetlands in northeastern North America (Saltonstall 2002, 2003). Over a dozen native haplotypes (*P. australis* subsp. *americanus* (Saltonstall et al. 2004); hereafter referred to as *americanus*) are found throughout North America (Saltonstall 2002), but haplotype E predominates wetlands in the northeastern US (Saltonstall 2011) and at our study sites (Saltonstall 2003).

The presence of adjacent populations of *australis* and *americanus* provides an important means of making comparative inferences about the nature of soil pathogen communities that may be unique to *australis* and hence may contribute to invasive success. First, because we do not know when *australis* populations were first introduced to our study sites, our paired site selection increases the likelihood that the soils did not differ in the composition of pathogen communities at the time of initial establishment, making the differences that we observe more likely to be driven solely by *Phragmites* colonization. Second, although the impacts of pathogen communities on plant performance were not evaluated directly in this study, large differences in the relative performance of *australis* and *americanus* were observed at our study sites. For example, individual plants within *australis* populations were taller, had larger seed heads, and produced more viable seed than those within *americanus* populations. Additionally, *australis* populations were denser, and individual patches were larger than those of *americanus* populations. Finally, in recent years, patch sizes of *australis* have been expanding at these sites, whereas those of *americanus* have been declining. Given that *australis* and *americanus* are genotypically nearly identical, any differences that we observe in soil pathogen communities will be highly correlated with performance of the two plant genotypes.

The aim of our study was to test the hypothesis that the structure of soil oomycete pathogen communities differs between non-native *australis* and native *americanus* populations, consistent with the predictions of PSF theory. Our aim was to focus directly on the oomycete communities recruited to either *australis* or *americanus* rhizospheres as a result of the long-term soil conditioning by populations of each respective haplotype. Our objectives were to (1) determine the species composition, species overlap, and phylogenetic similarity of pathogenic soil oomycetes in the rhizosphere of *americanus* and *australis* populations and (2) determine which specific oomycete taxa contributed most significantly to any differences observed. While our longer term goal is to assess how these specific changes in pathogen communities influence plant performance, the work reported here is meant to serve as a foundation for identifying specific candidate pathogens that may then be subsequently evaluated for their differential virulence to *australis, americanus,* and non-native plants at different stages of plant development as well as their populations dynamics that track with plant growth. With this additional knowledge of pathogen dynamics, more rational experiments can then be designed to assess the relative roles of different pathogens on plant performance.

## Material and Methods

### Study site and soil sampling

We identified four sites within and near the Montezuma National Wildlife Refuge that supported populations of both *americanus* and *australis*. Established populations of *americanus* and *australis* at each site were separated by ≤100 m, increasing the likelihood of similar microclimates, soil characteristics, pathogen communities, and plant communities. These four sites were designated as follows: (CC) Carncross, (EP) Eagle Point, (RR) Railroad, and (Rt31) US Route 31. Each of these sites contained mixed native and non-native plant communities. All *americanus* and *australis* populations were intermittently flooded, with the exception of the *australis* population at the CC site.

Rhizosphere soils were collected on 15 May and 15 December in 2008 and at 2-month intervals beginning mid-May 2009 and ending in mid-May 2010. This temporal sampling design was chosen to eliminate any seasonal phenological influences on the species composition and relative abundance of pathogenic taxa. However, the relatively small sample size at any one sampling time precluded any subsequent analyses of temporal effects on the distribution and density of individual taxa. Soils (∼40 g/sample) were collected with a 2.5-cm-diameter soil borer to a depth of 15 cm immediately adjacent (≤1 cm) to individual plants at five randomly chosen locations within the center of each population. Litter was removed prior to soil collection, and sampling locations within the population were stratified across sampling times. Individual soil samples were pooled for each population (∼200 g soil from each population), placed in plastic bags, and transported in a cooler back to the laboratory. Prior to soil DNA extractions, any root fragments, litter, and other debris were removed. DNA was then extracted immediately from each of the 8 soil samples (one for each of the four *americanus* populations and one for each of the *australis* populations) as outlined below.

### DNA extraction and PCR conditions

DNA was extracted using the Fast DNA® SPIN for Soil Kit (MP Biomedicals, LLC, Santa Ana, CA) from a 0.5 g soil sample on the day of soil collection. DNA was further purified with the Ultraclean™ PCR Clean-Up Kit (Mo Bio Laboratories, Inc., Carlsbad, CA) prior to PCR amplifications. Soils and DNA samples were then stored at −20 and −80°C, respectively, for archiving.

Polymerase chain reactions (PCRs) for DNA extracted directly from rhizosphere soils were completed using a nested approach. The primers 5.8SR and LR7 were used first to amplify approximately 1400 bp of the 28S region of the rRNA gene (Table S1). This was followed with the Oom1 and Sap2 primer sets described previously (Arcate et al. 2006). All PCR reactions contained 10 mmol/L Trizma HCl, pH 8.3, 50 mmol/L KCl, 2.5 mmol/L MgCl_2_, 0.2 *μ*mol/L of each primer, 200 *μ*mol/L of each dNTP, 1 unit of Sigma REDTaq Genomic Polymerase, and 0.5 *μ*L of template DNA per 25 *μ*L reaction. DNA was amplified with a Bio-Rad MyCycler™ (Bio-Rad Laboratories, Hercules, CA) thermal cycler using the appropriate PCR conditions listed in Supplementary Table S1.

### Cloning and sequencing

PCR products from rhizosphere soils were cloned using INV*α*F' competent cells and the pCR®2.1 vector from the TA Cloning® Kit (Life Technologies, Grand Island, NY) according to the manufacturer's directions. Transformants were transferred to Luria-Bertani (LB) broth containing kanamycin (50 *μ*g/mL) in 96-well tissue culture plates, incubated for 16 h at 37°C at 225 rpm, then screened with PCR using the M13f and M13r primers **(**Table S1**)** to check for the presence of an insert. Proper-sized transformants were purified with exonuclease 1 and antarctic phosphatase (New England BioLabs, Inc., Ipswich, MA) for 45 min at 37°C then at 95°C for 15 min prior to sequencing. Purified plasmid DNA was mixed with the M13f primer then submitted to the Cornell University Life Sciences Core Laboratories Center. Sequencing was performed on an Applied Biosystems Automated 3730 DNA Analyzer (Life Technologies) using Big Dye Terminator chemistry and AmpliTaq-FS DNA Polymerase. Sequences were compiled and edited in Sequencher 4.8 (Gene Codes Corp., Ann Arbor, MI) to remove vector sequences and eliminate poorly resolved regions. Sequence affinities to known taxa were determined based on BLAST searches of the NCBI GenBank database.

### Sequence alignments

Edited and trimmed sequences were imported into MEGA 5.0 (Tamura et al. 2011) and aligned using the MUSCLE algorithm (Edgar 2004) under the default settings. Ribosomal DNA sequences from reference taxa were included in some alignments used for subsequent phylogenetic analysis. After initial alignments, sequences were manually edited using MEGA 5.0 to correct misaligned sequences and ambiguous base designations. During this final editing, all alignments were further trimmed to a fixed length. For subsequent phylogenetic analyses, only those reference sequences with apparent affinities to our unknown sequences were included in the alignment.

### Estimation of community species richness

Jukes–Cantor-corrected distance matrices were constructed from combined Oom1 and Sap2 alignments for individual plant populations using the DNAdist utility within the Phylip v3.68 program. These distance matrices were then used to estimate species richness of each library using rarefaction analysis as implemented in DOTUR (Schloss and Handelsman 2005). To estimate the most appropriate operational taxonomic unit (OTU), cutoff values for our analyses, distance matrices were also constructed from alignments of 28S rRNA gene sequences from selected oomycete species retrieved from GenBank. Accessions were chosen from what we considered to be morphologically well-characterized species from the Centraalbureau voor Schimmelcultures (CBS) collection. These distance matrices were analyzed using DOTUR to determine the distance level that accurately predicted the number of OTUs in the alignment. Based on these results, species-level OTUs were defined at a distance level of 0.03. Consensus sequences of OTUs used in subsequent phylogenetic analyses were generated in Megalign 10.0.0 (Lasergene, DNASTAR, Inc., Madison, WI). The relatedness of each of our calculated OTUs to known taxa was determined using BLAST searches of the GenBank database. As it was not clear whether singletons or doubletons (sequences detected only once or twice) truly represented very rare taxa or were simply artifacts of the cloning, PCR, and sequencing, they were eliminated from all community analyses.

### Phylogenetic estimates of oomycete community similarity

Phylogenetic relationships of oomycete taxa among the 8 *Phragmites* populations and between the combined *americanus* and *australis* populations were analyzed using the neighbor-joining method of tree construction (Saitou and Nei 1987) as implemented in MEGA 5.0. Phylogenies were then used as the basis for estimating similarity among oomycete communities associated with the *Phragmites* populations. The level of OTU overlap among oomycete communities was estimated using the SONS software package (Schloss and Handelsman 2006). Both qualitative unweighted (presence/absence of OTUs) and quantitative weighted (relative abundance of OTUs) estimations of community similarity were used to calculate UniFrac distance metrics as implemented in the Fast UniFrac software platform (Hamady et al. 2010). Because of the uneven sequence sampling across soils from *americanus* and *australis* populations, all OTU abundances from each population were normalized relative to the sampling effort within that population so that relative abundances were expressed as a proportion of the total sampling effort for that population. Pairwise community comparisons from individual and combined *americanus* and *australis* populations were assessed, and the significance of community differences determined using a Monte Carlo simulation by randomly assigning community membership to sequences and estimating *P*-values based on 1000 random sample permutations.

The *P*-test (Martin 2002), which estimates similarity between communities as the number of parsimony changes required to explain the distribution of sequences between one community or the other in a phylogenetic tree, was used as an additional metric of community similarity. The statistical significance of differences (based on a *P*-value) represents the fraction of 1000 random permutations of the terminal taxa that require fewer parsimony changes to explain than does the actual tree. All *P*-values were corrected for multiple comparisons using the Bonferroni correction. Community similarity/dissimilarity was ordinated using a principal coordinate analysis (PCoA) based on Jukes–Cantor-corrected distance matrices. A generalized Morisita index of community similarity (Chao et al. 2006) was also used to estimate the level of oomycete community similarity between soils from *americanus* and *australis* populations. This index was calculated using SPADE (Chao and Shen 2003).

Relative oomycete OTU abundances among the different *americanus* and *australis* populations were then subjected to similarity percentage (SIMPER) analysis (Clarke 1993) to determine which OTUs contributed most to the observed community dissimilarity. To compare communities, the Bray–Curtis dissimilarity measure was used as implemented in the software program PAST v. 2.10 (Hammer et al. 2001). Percent community dissimilarity of each pairwise comparison was calculated, and the percent contribution of each OTU to the overall dissimilarity between each of the pairwise community comparisons was determined.

## Results

### OTU richness of *americanus* and *australis* soil oomycete communities

**Figure 1:**
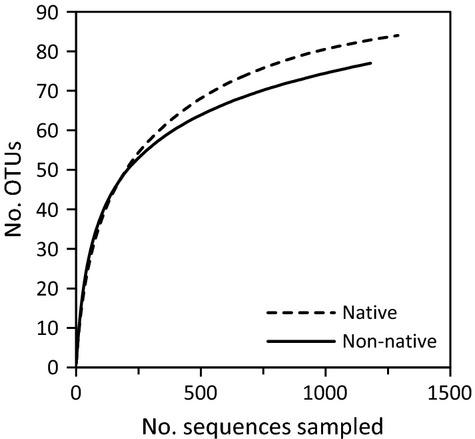
Species richness of soil oomycete communities from *americanus* and *australis* populations (from all four wetland sites combined) based on rarefaction analysis using the DOTUR software package. Species richness did not differ (*P* = 0.05) between soil communities from *americanus* and *australis* populations.

Over a 24-month period, between 139 and 423 oomycete sequences (=individuals) were sampled from rhizosphere soils of each of the *americanus* and *australis* populations **(**Table S2**)** for a total of combined population size of 1271 and 1168 individuals from *americanus* and *australis* populations, respectively. This level of sampling yielded between 25 and 39 OTUs (*d* = 0.03) among the four *americanus* populations and between 15 and 35 OTUs among four *australis* populations. Although oomycete species richness among the combined *americanus* populations did not differ from the combined *australis* populations **(**Fig. 1), significantly fewer total OTUs were detected in the rhizospheres of *australis* populations than in the *americanus* populations at the EP and CC sites (Supplementary Fig. S1). No differences were detected in the number of OTUs between *americanus* and *australis* populations at the RR and Rt31 sites.

### Oomycete taxa associated with *americanus* and *australis* rhizospheres

**Figure 2:**
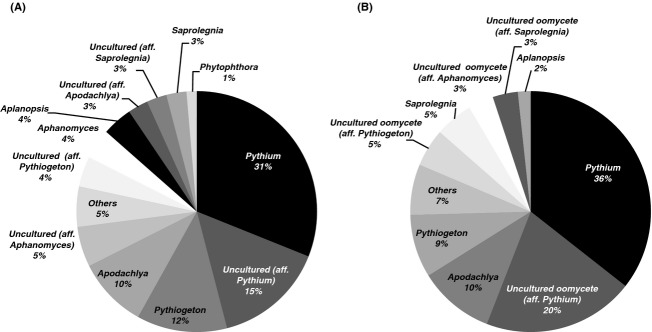
Distribution of the major genera of oomycetes detected in soils from *americanus* (A) and *australis* (B) populations. Genera in the “Others” category include *Dictyuchus, Halioticida, Leptolegnia*, and *Leptomitus*. Singleton and doubleton sequences were eliminated from the distribution.

**Figure 3:**
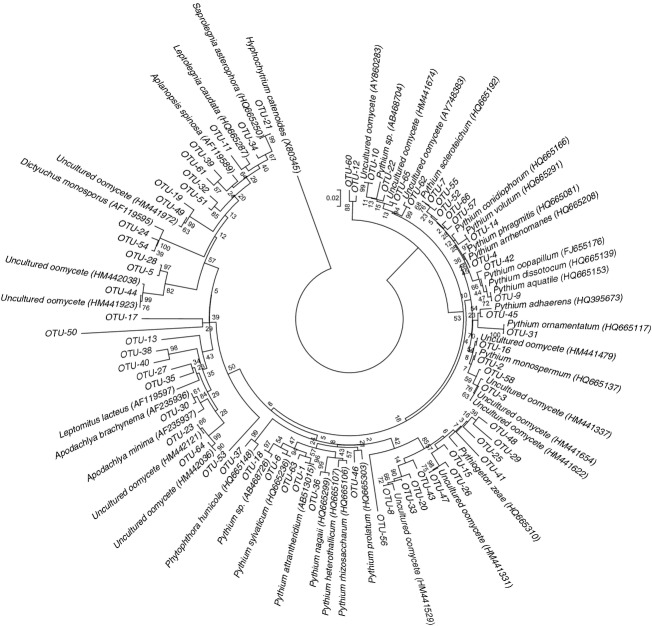
Phylogenetic relationships of oomycete taxa recovered from soils associated with *americanus* and *australis* populations. The relationships among taxa were inferred using the neighbor-joining method. The optimal tree with the sum of branch length = 2.06340903 is shown. The tree is drawn to scale, with branch lengths in the same units as those of the evolutionary distances used to infer the phylogenetic tree. The evolutionary distances were computed using the Jukes–Cantor method and are in the units of the number of base substitutions per site. The analysis involved 108 nucleotide sequences. All positions containing gaps and missing data were eliminated. There were a total of 338 positions in the final dataset. Evolutionary analyses were conducted in MEGA5.

Oomycete taxa associated with both *americanus* and *australis* rhizospheres were dominated by species of *Pythium* and uncultured oomycetes with affinities to *Pythium* species **(**Table S3, Fig. 2A,B), most of which are known plant pathogens of a broad range of plant species (Table S4). There was considerable variation in the dominant taxa from site to site (Fig. S2), with some oomycete taxa detected at one site but not at the others. Over 62% of all the oomycete sequences detected were recovered from *australis* soils whereas just over 42% were recovered from *americanus* soils. Although many of the most abundant OTUs had clear affinities to *Pythium* species, many other OTUs remain unknown, despite their phylogenetic placement solidly within the genus *Pythium* and *Pythiogeton* (Fig. 3).

### Oomycete community dissimilarities between *americanus* and *australis* populations

**Figure 4:**
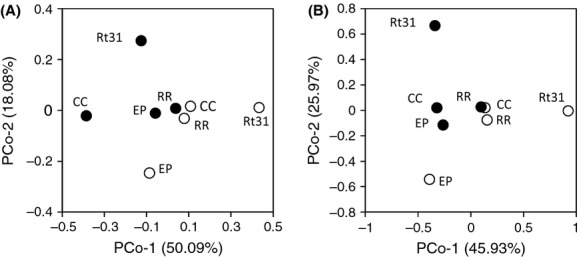
Principal coordinate analyses (PCoA) of soil oomycete communities associated with *americanus* (open circles) and *australis* (black circles) populations. CC, Carncross site; EP, Eagle Point site; RR, Railroad site; Rt31, Route 31 site. The analyses were based on Jukes–Cantor-derived distance matrices calculated using either presence/absence (A) or relative abundance (B) of oomycete taxa. Numbers in parentheses on each PCo axis represent the percentage of the total variance explained by that PCo. Only the first two principle components are shown.

Phylogenetic comparisons of oomycete communities from *americanus* and *australis* populations revealed significant community differences between individual sites and between combined *americanus* and *australis* populations, regardless of whether comparisons were based on presence/absence or relative abundance of individuals. Based on unweighted (OTU presence/absence) and weighted (OTU relative abundance) UniFrac metrics, the P-test metric, and the Morisita index of similarity, both the composition and relative abundance of OTUs from *americanus* populations were significantly different from communities associated with *australis* populations (Table 1). The only exceptions to this were the RR oomycete communities where the Morisita index indicated a greater level of similarity than dissimilarity between *americanus* and *australis* populations. Additional analyses using both weighted and unweighted principal coordinate analysis indicated that oomycete communities from *australis* populations clustered from those of *americanus* populations (Fig. 4). Ordination of these communities across the first two principal coordinate explained over 70% of the variation observed in the community dataset.

**Table 1:** Statistical comparisons of oomycete species composition and relative abundance between *americanus* and *australis* populations at four different sites

	Oomycete community dissimilarity between *americanus* and *australis* populations at four wetland sites		
	
Site	*P*-Test^1^	Unifrac test^2^	Morisita similarity index (±95% CI)^3^
CC	<0.001	<0.001	0.020 (±0.019)^1^
EP	<0.001	<0.001	0.295 (±0.066)^1^
RR	<0.001	<0.001	0.627 (±0.134)
Rt31	<0.001	<0.001	0.414 (±0.086)^1^
Combined sites	<0.001	<0.001	0.410 (±0.058)^1^

1 *P*-test estimates similarity between communities as the number of parsimony changes required to explain the distribution of sequences between one community of the other in the phylogenetic tree. The statistical significance of the differences (expressed as a *P*-value) represents the fraction of 1000 random permutations of the terminal taxa that require fewer parsimony changes to explain the phylogeny than does the actual tree.

2 Unifrac metric Lozupone et al. (2007) measures the difference between two communities by comparing the phylogenetic tree branch lengths unique to one community or the other. Both the unweighted (presence/absence only) and weighted (branch lengths are weighted based on the relative abundance of individual OTUs in the respective communities) Unifrac metric was used and yielded the same result. To test the significance of these differences, community affiliations (native or non-native) were randomly permuted (1000 times) across sequences in the tree and new UniFrac values calculated each time. Communities were considered to be significantly different if the UniFrac values for the real community phylogenies were greater than would be expected if the sequences were randomly distributed between the two communities. The reported *P*-value is the fraction of permuted trees that have UniFrac values greater or equal to that of the real phylogenetic tree.

3 Morisita index was used to assess community similarity based on abundance data of OTUs. Estimated using the software SPADE Chao and Shen (2003). Numbers in parentheses represent 95% confidence intervals for the estimate. Indices followed by an asterisk indicate that *americanus* and *australis* communities were significantly (*P* = 0.05) different.

**Figure 5:**
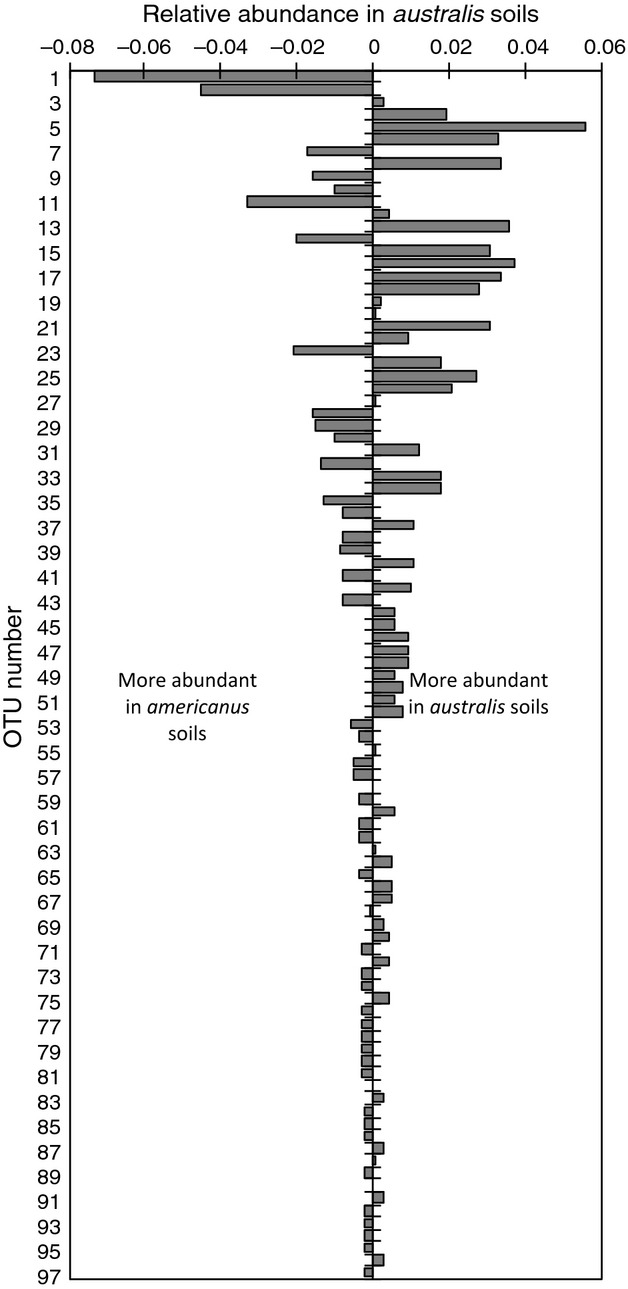
Relative abundance of OTUs from the combined *americanus* and combined *australis* wetland sites providing the greatest contributions to overall oomycete community differences between soils from *americanus* and *australis* populations as determined by SIMPER analysis using Bray–Curtis distance measures. Relative abundance of each OTU is proportional to the contribution that each OTU makes to the overall differences between *americanus* and *australis* oomycete communities. Overall dissimilarity of oomycete communities between *americanus* and *australis* populations was 76.97%. Refer to Table S3 for OTU affiliations.

The 25 and 50 most abundant OTUs collectively explained 70% and 89% of the observed differences in relative abundance of oomycete taxa between *americanus* and *australis* populations, respectively. In general, the OTUs contributing most significantly to oomycete community dissimilarities were equally distributed between *australis* and *americanus* soils (Fig. 5). However, OTUs in *australis* soils that had the greatest influence on the observed community differences were distinct from those in *americanus* soils. At any individual site, however, there was considerable variation in the specific OTUs that contributed most to the observed oomycete community differences (Fig. S3). Despite this variation, some OTUs were consistently found in *americanus* soils (e.g., OTU-1 (*P. attrantheridium*), OTU-2 (*P. monospermum*), and OTU-7 (*P. scleroteichum*)), whereas others were found more consistently in *australis* soils (e.g., OTUs 3 and 16 (uncultured oomycetes aff. *Pythium*)). The levels of oomycete community dissimilarity at each of the sites were 97.8, 78.1, 53.1, and 69.0%, respectively, for the CC, EP, RR, and Rt31 sites. These results mirrored those obtained using Morisita estimates of similarity/dissimilarity.

## Discussion

Our results show a clear distinction between the oomycete pathogen communities associated with *australis* and *americanus* soils, differing not only in species composition but in the relative abundances of specific taxa. Although our methodologies were designed to detect the full breadth of the oomycete phylogeny, our comparative assessments revealed a particularly high prevalence *Pythium* species associated with both *americanus* and *australis* soils. Species within this genus are broad host-range pathogens of many plant species, and many of the species we detected are important pathogens of the Poaceae. The dominance of *Pythium* species in these wetland sites is not surprising, given the wide distribution of *Pythium* in soils from both natural and agricultural ecosystems (Martin and Loper 1999; Gilbert 2002). However, the prevalence and distribution of specific species such as *P. attrantheridium* and *P. sylvaticum* were not entirely expected as neither species has been described from wetland soils, and *P. attrantheridium* in particular has rarely been found as a dominant species in any soil and may even prefer dryer soils (Schroeder et al. 2006).

Our work sought to determine whether communities of oomycete pathogens associated with *americanus* populations in noninvaded soils differed from those associated with *australis* populations in invaded soils. This was based largely on an untested prediction of PSF theory, implicating changes in communities of oomycete pathogens to differential negative PSFs and invasive success (Klironomos 2002; Reinhart et al. 2005, 2010, 2011; Callaway et al. 2011). To explain invasive success of *australis*, pathogen-induced negative PSFs experienced by native noninvasive and rare species, including *americanus*, should be greater than those experienced by invasive and dominant species (Klironomos 2002; van Grunsven et al. 2007). For this to occur (assuming PSFs were all direct and not indirectly mediated through other agents), we would expect to observe either (1) greater pathogen richness, (2) greater abundance of specific pathogenic taxa (Mills and Bever 1998; Westover and Bever 2001), or (3) a higher level of virulence among pathogenic taxa (Reinhart et al. 2010) in *americanus* than in *australis* soils. Our results are consistent with this prediction as very different assemblages of oomycete pathogens were observed in soils conditioned by *americanus* and *australis* populations. Although species richness did not differ between *americanus* and *australis* soils, some pathogens such as *P. attrantheridium, P. monospermum, P. scleroteichum,* and *P. volutum* were all more abundant in *americanus* soils than in *australis* soils. Therefore, if these species contribute to *australis* dominance at our sites, infection of *americanus* individuals by these pathogens should reduce performance and competitiveness relative to *australis* individuals. While this is possible as *P. attrantheridium* and *P. volutum* have been implicated previously in negative PSFs that limit the dominance of plant species (Mills and Bever 1998; Packer and Clay 2004; Reinhart et al. 2010), their involvement in our system remains unclear, in part, because the virulence of these taxa to *americanus* or *australis* individuals and their impacts on performance are unknown.

The interpretation above is further complicated by observations of other important pathogenic taxa, such as *Pythium sylvaticum* and *P. phragmitis*, that dominate *australis* soils. *Pythium sylvaticum* is potentially significant because of its demonstrated role in negative PSFs with other plant species and its strong regulatory role in limiting the dominance of a forest invasive species in its native range (Reinhart and Clay 2009; Reinhart et al. 2010, 2011). However, beyond these studies, little is known of its distribution and functional significance in natural ecosystems.

Equally significant is the detection of *Pythium phragmitis* in *australis* soils. Wetland soils from the native European range of *australis* are commonly dominated by *Pythium phragmitis* (Nechwatal et al. 2005) and other diverse oomycete pathogens (Nechwatal et al. 2005, 2008a), including many that are virulent to *Phragmites* seedlings (Nechwatal et al. 2008a,b; Nechwatal and Mendgen 2009) and other members of the Poaceae. If *americanus* seedlings are differentially more susceptible to *P. phragmitis, P. sylvaticum*, and other pathogenic taxa than *australis* seedlings, any pathogen amplification during soil conditioning by *australis* populations may limit the establishment and subsequent dominance of *americanus* seedlings and possibly other native species in invaded sites.

These two different interpretations suggest multiple hypotheses for how plant–pathogen interactions may potentially facilitate invasive success. For example, for pathogens that dominate *americanus* soils, it is possible that these pathogens may limit the demographic expansion of *americanus* and other native plant populations not affect *australis* populations to the same degree; a mechanism commonly embodied by the enemy-release concept (Keane and Crawley 2002; van Grunsven et al. 2007). Alternatively, pathogens in *americanus* soils may affect *americanus* and *australis* populations equally as might be expected with phylogenetically close, if not identical, plant species (Gilbert and Webb 2007). However, *australis* populations may be able to engineer positive PSFs for themselves that override any pathogen-induced negative PSFs they may experience (Inderjit and van der Putten 2010), providing a greater net negative PSF experienced by *americanus* populations. In contrast, pathogens in *australis* soils may accumulate and spillback to *americanus* and other native plant populations reducing their competitiveness with *australis* populations (Eppinga et al. 2006; Mangla et al. 2008; Flory et al. 2011).

Our results point to other hypotheses for soil pathogen invasion facilitation. The strikingly lower abundance of some pathogenic taxa in *australis* soils (e.g., *P. attrantheridium*, *P. monospermum*, and *P. scleroteichum*) may be evidence for a selective inhibition of certain taxa by *australis* populations, again allowing *australis* to potentially escape infection. *Pythium attrantheridium* in particular has a broad host range (Allain-Boule et al. 2004), but commonly associated with other grasses (Schroeder et al. 2006) such as *australis*. Although some plant roots produce microbial inhibitors upon infection by oomycete pathogens (Bais et al. 2003, 2005), some non-native invasive species produce root exudate molecules that inhibit oomycete pathogens (Zhang et al. 2009, 2011). Such inhibitory molecules from *australis* roots have not been described, but this may be a reasonable hypothesis to explain the oomycete community patterns we observed.

An important consideration for establishing specific oomycete pathogens with negative PSFs and invasive success is the nature of the differential host responses required for one plant population to dominate over another. It is commonly assumed that specialist host-specific pathogens are central to the establishment of such differential negative PSFs and host responses (Bever et al. 2012). However, these inferences have been made largely from observations only of *host*-specific responses and not from observations of *pathogen*-specific community responses that accompany host changes. Our results have revealed a diverse community consisting largely of generalist broad host-range *Pythium* species (Richardson et al. 2000; Augspurger and Wilkinson 2007) that can also persist saprobically in soil (Lockwood 1988). If soil pathogens are central to negative PSFs and invasive success, they are likely to be generalist rather than host-specific soil pathogens.

There are several ways in generalist pathogens could give rise to host-specific responses. For example, individual isolates or strains within a pathogen population often vary greatly in their virulence to a given host species (Augspurger and Wilkinson 2007; Reinhart et al. 2010; Vachon and Freeland 2011). Depending on the complex of virulence phenotypes within the pathogen population and among pathogenic species within a community, different spectra of plant responses would be expected. This is why inferences based on the isolation of only a single isolate or a small number of isolates within a given pathogenic species may not accurately predict host responses. Additionally, there is often considerable individual variation in host resistance and tolerance to a given pathogen within a plant population and within and between phylogenetically related species (Jarosz and Davelos 1995; Gilbert and Webb 2007). This variation alone could explain differing levels of host-specific responses in the presence of generalist soil pathogens. However, there are other indirect ways in which differential host responses to generalist pathogens could be achieved.

Other plant-associated microbes may modulate host defenses to pathogens in very host-specific ways. Different plant species and genotypes are known to amplify and recruit unique bacterial and fungal communities to the rhizosphere (Westover et al. 1997; Kowalchuk et al. 2002; Vandenkoornhuyse et al. 2003; Pendergast et al. 2013), some of which are known to suppress plant infections by pathogens (Berendsen et al. 2012; Latz et al. 2012). Such an interaction may also provide *australis* populations with positive PSFs that could offset any pathogen-induced negative PSFs, leaving *americanus* populations more negatively affected. Furthermore, soil edaphic factors and other environmental variables may also influence host susceptibility (Hersh et al. 2012) and/or broad soil microbial assemblages (Lauber et al. 2008), both of which could either offset negative PSFs induced by pathogens or even exacerbate already established negative PSFs.

It is clear that PSFs experienced by introduced non-native species are not static and change with increasing residence time in the introduced range (Hawkes 2007; Diez et al. 2010), largely because of the accumulation of pathogens over time (Mitchell et al. 2010; Phillips et al. 2010). This too further complicates interpretation of the specific pathogens that may facilitate invasive success. Our assessments of pathogen communities were made from soils collected at a rather advanced stage of an invasion where *australis* populations were present in extensive well-established patches. It is possible that any pathogens detected at this stage of the invasion process could have little to do with initial invasive success if the important negative PSFs occurred earlier. Had we sampled soils shortly after seed introduction and seedling establishment, we likely would have observed a very different assemblage of oomycete pathogens that would have been critical in the initial establishment of new invasion foci. Others have stressed the importance of examining pathogen dynamics over the course of an invasion (Flory and Clay 2013). However, this is especially challenging for *Phragmites* invasions because of their cryptic initiation from small, difficult-to-observe seedlings, making the dating and early progression of an invasion difficult to determine. It is possible, however, to sample communities from seeds buried in soil or at the advancing edge of an expanding patch, both of which may reveal other unique oomycete community differences between *australis* and *americanus* populations that would establish important patterns related to invasive success.

It is clear from our work here that definitive inferences about the role of specific soil pathogens cannot be made without comprehensive assessments of virulence and host range from rather large sampling of isolates from both *americanus* and *australis* populations. Even with the clear identification of highly virulent and weakly virulent species, a number of challenges remain in determining specific pathogen populations that contribute to invasive success. For example, not only are multiple pathogens commonly recovered from individual plants, but both virulent and avirulent strains of the same pathogen species may coexist in the rhizosphere (Gomez-Alpizar et al. 2011). These diverse pathogen populations may compete with each other (Vandemark et al. 2010), allowing some nonpathogenic species to induce positive PSFs by suppressing primary pathogenic species (Vallance et al. 2009), whereas other co-occurring species may have no apparent impact on primary pathogens (Suffert and Guibert 2007). A more detailed understanding of the distribution and dynamics of pathogens, nonpathogens, and host-associated mutualists and commensals will provide greater insights for understanding plant–microbe interactions, and the roles they play in plant community dynamics and plant invasions.

## Data Accessibility

Selected sequences corresponding to each of the described OTUs have been submitted to GenBank under accession numbers KF745952 to KF746017.

## Supporting Information

**Figure S1.** Species richness of soil oomycete communities from *americanus* and *australis* populations at each of four wetland sites based on rarefaction analysis using the DOTUR software package: (A) Carncross site, (B) Eagle Point site, (C) Railroad site, (D) Rt 31 site.

**Figure S2.** Distribution of OTUs among *americanus* (A) and (B) *australis* populations as determined by SONS analysis.

**Figure S3.** Relative abundance of OTUs from each of the four *americanus* and *australis* wetland sites providing the greatest contributions to overall oomycete community differences between soils from *americanus* and *australis* populations as determined by SIMPER analysis using Bray-Curtis distance measures.

**Table S1.** Primers and associated PCR conditions utilized in this study.

**Table S2.** General properties of sequence libraries from native and non-native *P. australis* rhizospheres in each of four wetland sites.

**Table S3.** Taxon affiliations of OTUs detected in rhizosphere soils associated with *americanus* and *australis* populations, ordered from most abundant to least.*

**Table S4.** Reported plant host ranges of oomycete taxa detected in soils from *americanus* and *australis* populations.
