# Supplementary figures and images for: Soil pathogen communities associated with native and non-native Phragmites australis populations in freshwater wetlands
Source: Ecol Evol. 2013 Dec 3;3(16):5254–67. doi: 10.1002/ece3.900 (PMC3892333; doi:10.1002/ece3.900)

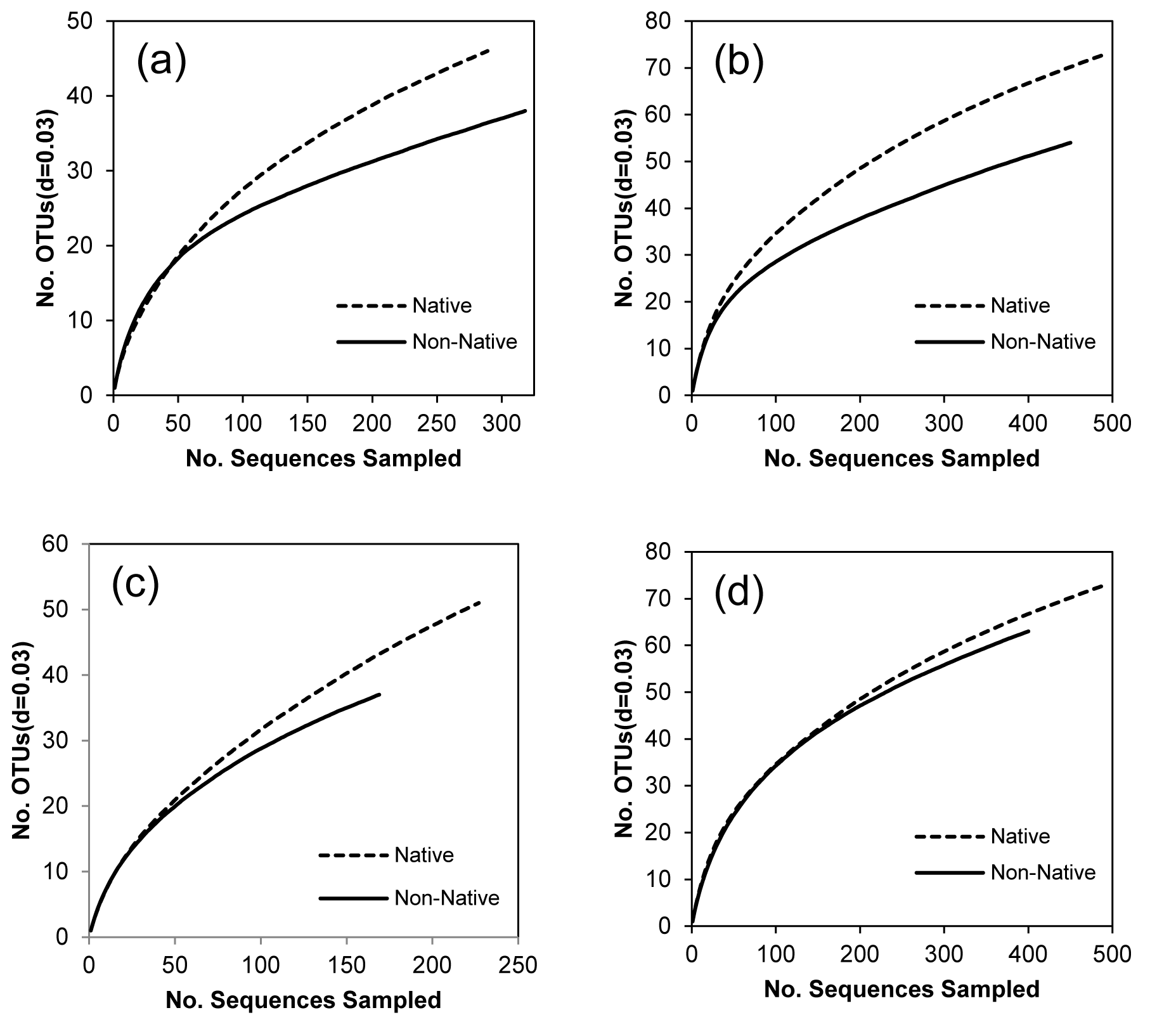

Supplement: Supplementary file 1 [file ece30003-5254-SD1.tif]

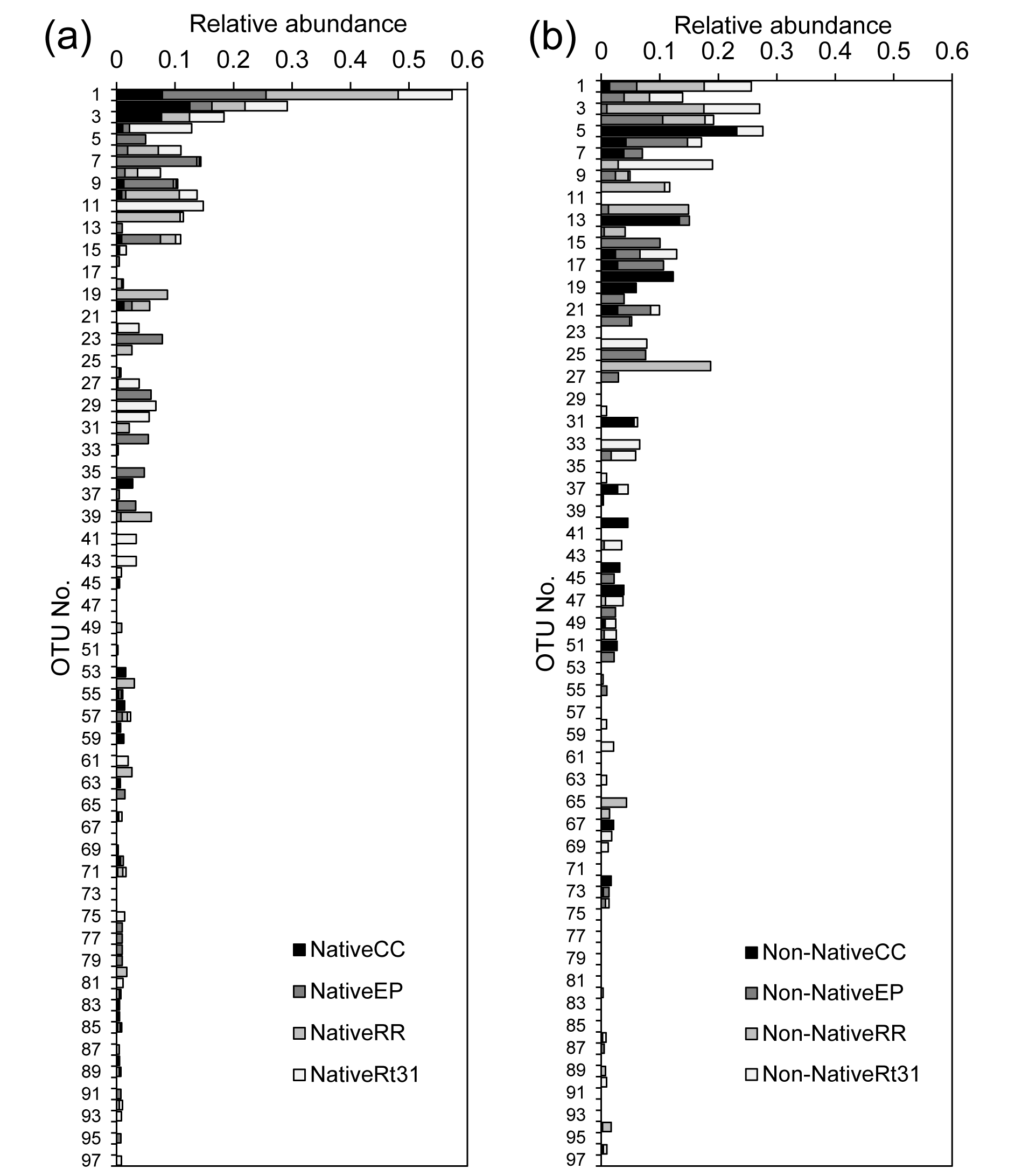

Supplement: Supplementary file 2 [file ece30003-5254-SD2.tif]

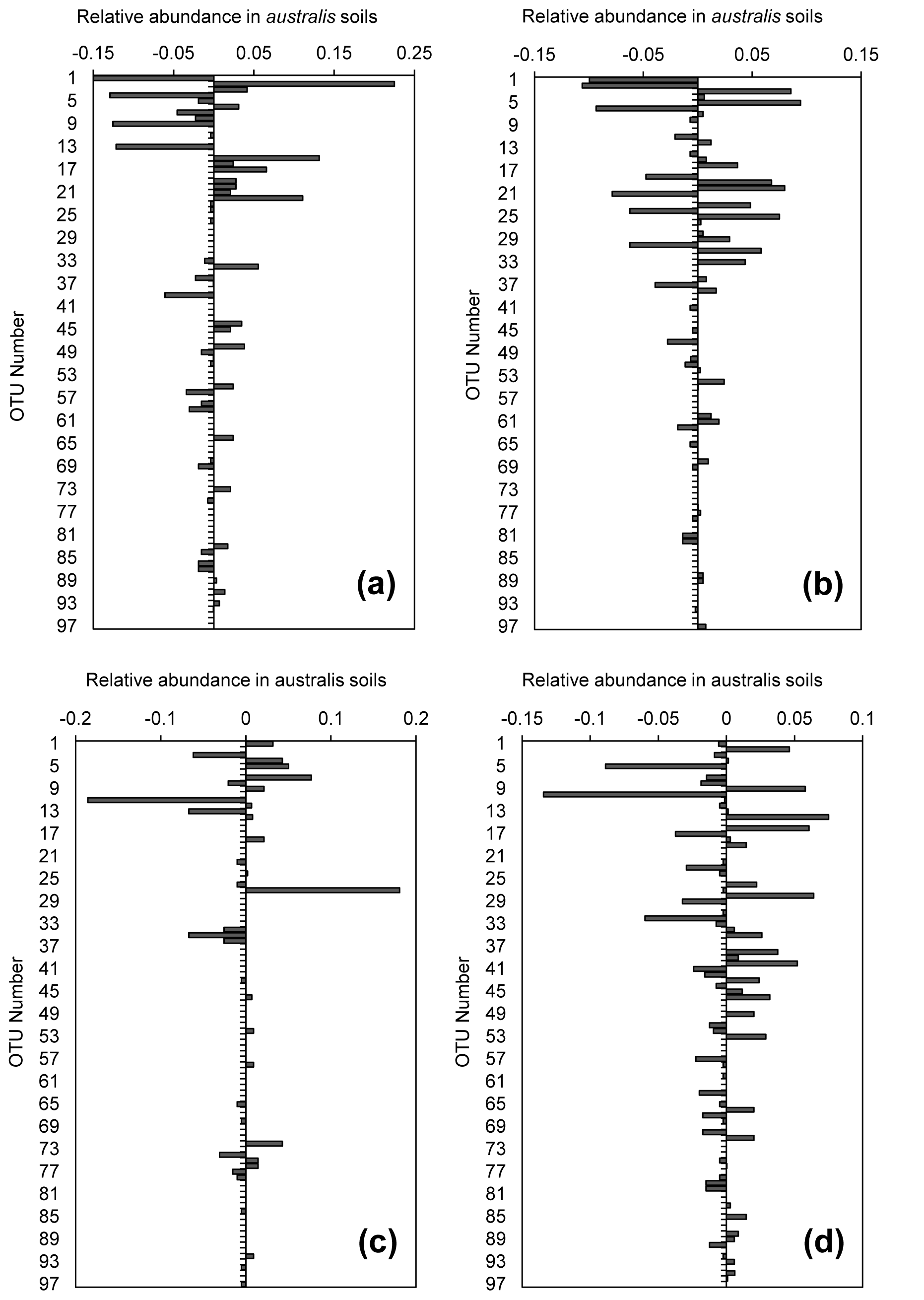

Supplement: Supplementary file 3 [file ece30003-5254-SD3.tif]
