# Supplementary material for: Soil pathogen communities associated with native and non-native Phragmites australis populations in freshwater wetlands
Source: Ecol Evol. 2013 Dec 3;3(16):5254–67. doi: 10.1002/ece3.900 (PMC3892333; doi:10.1002/ece3.900)
Supplement: Supplementary file 4 [file ece30003-5254-SD4.pdf]

Table S1. Primers and associated PCR conditions utilized in this study

Primer Set	Primers	Primer Sequence	PCR Conditions	Reference
1	5.8 SR<br>LR7	5'-TCGATGAAGAACGCAGCG -3'<br>5'-TACTACCACCAAGATCT-3'	Initial denaturation: 94˚C for 5 min, 30 cycles, denaturation: 94˚C for 30 s, annealing: 47˚C for 90 s, extension: 72˚C for 60 s (10 min final extension)	(Vilgalys & Hester 1990)
2	Oom1f<br>Oom1r	5'-GTGCGAGACCGATAGCGAACA-3'<br>5'-TCAAAGTCCCGAACAGCAACAA-3'	Initial denaturation: 94˚C for 5 min, 35 cycles, denaturation: 94˚C for 30 s, annealing: 58.4˚C for 30 s, extension: 72˚C for 30 s (10 min final extension)	(Arcate, Karp & Nelson 2006)
3	Sap2f<br>Sap2r	5'-AGCATAGCGATTTGGGATAAGTC-3'<br>5'-GTAGGCACCTCAGTCTCAACCA-3'	Initial denaturation: 94˚C for 5 min, 35 cycles, denaturation: 94˚C for 30 s, annealing: 54.4˚C for 30 s, extension: 72˚C for 30 s (10 min final extension)	(Arcate, Karp & Nelson 2006)
4	M13f<br>M13r	5'-GTAAAACGACGGCCAG-3'<br>5'-CAGGAAACAGCTATGAC-3'	Initial denaturation: 94˚C for 5 min, 30 cycles, denaturation: 94˚C for 30 s, annealing: 50˚C for 30 s, extension: 72˚C for 30 s (10 min final extension)	(Huang *et al.* 2013)

References

Arcate, J.M., Karp, M.A. & Nelson, E.B. (2006) Diversity of peronosporomycete (oomycete) communities associated with the rhizosphere of different plant species. *Microbial Ecology,* **51,** 36-50.

Huang, J.-H., Chen, C.-Y., Lin, Y.-S., Ann, P.-J., Huang, H.-C. & Chung, W.-H. (2013) Six new species of *Pythiogeton* in Taiwan, with an account of the molecular phylogeny of this genus. *Mycoscience,* **54,** 130-147.

Vilgalys, R. & Hester, M. (1990) Rapid genetic identification and mapping of enzymatically amplified ribosomal DNA from several *Cryptococcus* species. *Journal of Bacteriology,* **172,** 4238-4246.
