# Supplementary material for: Soil pathogen communities associated with native and non-native Phragmites australis populations in freshwater wetlands
Source: Ecol Evol. 2013 Dec 3;3(16):5254–67. doi: 10.1002/ece3.900 (PMC3892333; doi:10.1002/ece3.900)
Supplement: Supplementary file 5 [file ece30003-5254-SD5.pdf]

Table S2. General properties of sequence libraries from native and non-native *P. australis* rhizospheres in each of four wetland sites

Site	Origin	Total No. Sequences	Total OTUs*	Shared OTUs*
CC	*americanus*	260	25	2
	australis	285	22	
EP	*americanus*	423	32	11
	australis	409	31	
RR	*americanus*	230	30	12
	australis	139	15	
Rt31	*americanus*	358	39	10
	australis	335	35	

*Unique singletons and doubletons removed from comparative analyses. Data generated with the software Spade (Chao & Shen 2003). A total of 2439 sequences were analyzed.

Reference

Chao, A. & Shen, T.-J. (2003) SPADE (Species Prediction And Diversity Estimation). Program and User's Guide published at http://chao.stat.nthu.edu.tw.
