# Supplementary material for: Soil pathogen communities associated with native and non-native Phragmites australis populations in freshwater wetlands
Source: Ecol Evol. 2013 Dec 3;3(16):5254–67. doi: 10.1002/ece3.900 (PMC3892333; doi:10.1002/ece3.900)
Supplement: Supplementary file 6 [file ece30003-5254-SD6.pdf]

Table S3. Taxon affiliations of OTUs detected in rhizosphere soils associated with *americanus* and *australis* populations, ordered from most abundant to least.*

OTU	Closest Known Taxon† (GenBank Accession Number)	OTU	Closest Known Taxon† (GenBank Accession Number)	OTU	Closest Known Taxon† (GenBank Accession Number)
1	*Pythium attrantheridium (AB513015)*	34	*Saprolegnia anisospora (AF119609)*	66	*Pythium arrhenomanes (HQ665208)*
2	*Pythium monospermum (HQ665137)*	35	*Leptomitus lacteus (AF119597)*	67	*Leptomitus lacteus (AF119597)*
3	*Uncultured oomycete (HM441622) (aff. Pythium)*	36	*Pythium nagaii (HQ665299)*	68	*Pythium rhizosaccharum (HQ665106)*
4	*Pythium phragmitis (HQ665081)*	37	*Phytophthora humicola (HQ665148)*	69	*Uncultured oomycete (HM441669) (aff. Pythium)*
5	*Uncultured oomycete (HM442038) (aff. Aphanomyces)*	38	*Apodachlya minima (AF235937)*	70	*Apodachlya brachynema (AF235936)*
6	*Pythium sylvaticum (HQ665236)*	39	*Aphanomyces astaci (AF235940)*	71	*Pythium oopapillum (FJ655176)*
7	*Pythium scleroteichum (HQ665192)*	40	*Apodachlya brachynema (AF235936)*	72	*Saprolegnia asterophora (HQ665250)*
8	*Uncultured oomycete (HM441529) (aff. Pythiogeton)*	41	*Pythiogeton zeae (HQ665310)*	73	*Pythium phragmitis (HQ665081)*
9	*Pythium aquatile (HQ665153)*	42	*Pythium dissotocum (HQ665139)*	74	*Apodachlya minima (AF235937)*
10	*Uncultured oomycete (AY860283) (aff. Saprolegnia)*	43	*Pythiogeton zeae (HQ665310)*	75	*Saprolegnia asterophora (HQ665250)*
11	*Leptolegnia caudata (HQ665287)*	44	*Uncultured oomycete (HM441923) (aff. Aphanomyces)*	76	*Apodachlya minima (AF235937)*
12	*Pythium sp. (AB468704)*	45	*Pythium adhaerens (HQ395673)*	77	*Aphanomyces piscicida (AF235941)*
13	*Apodachlya minima (AF235937)*	46	*Uncultured oomycete (HM441474) (aff. Pythium)*	78	*Aplanopsis spinosa (AF119589)*
14	*Pythium volutum (HQ665291)*	47	*Uncultured oomycete (HM441331) (aff. Pythiogeton)*	79	*Saprolegnia unispora (HQ665152)*
15	*Pythiogeton zeae (HQ665310)*	48	*Pythiogeton zeae (HQ665310)*	80	*Pythium scleroteichum (HQ665192)*
16	*Uncultured oomycete (HM441479) (aff. Pythium)*	49	*Uncultured oomycete (HM441972) (aff. Saprolegnia)*	81	*Pythium sp. (AB468752)*
17	*Apodachlya brachynema (AF235936)*	50	*Halioticida noduliformans (AB506706)*	82	*Uncultured oomycete (HM441389) (aff. Pythium)*

18	*Pythium sp. (AB468726)*	51	*Aplanopsis spinosa (AF119589)*	83	*Uncultured oomycete (HM442033) (aff. Aphanomyces)*
19	*Uncultured oomycete (HM441972) (aff. Saprolegnia)*	52	*Pythium myriotylum (HQ665176)*	84	*Uncultured oomycete (HM441474) (aff. Pythium)*
20	*Uncultured oomycete (HM441529) (aff. Pythiogeton)*	53	*Pythium prolatum (HQ665303)*	85	*Pythium porphyrae (HQ665218)*
21	*Saprolegnia asterophora (HQ665250)*	54	*Dictyuchus monosporus (AF119595)*	86	*Uncultured oomycete (AY860283) (aff. Pythium)*
22	*Uncultured oomycete (HM441474) (aff. Pythium)*	55	*Pythium rhizo-oryzae (HQ665087)*	87	*Pythium monospermum (HQ665137)*
23	*Uncultured oomycete (HM442121) (aff. Apodachlya)*	56	*Pythium heterothallicum (HQ665107)*	88	*Uncultured oomycete (HM441417) (aff. Pythium)*
24	*Dictyuchus monosporus (AF119595)*	57	*Pythium sp. (AF119609)*	89	*Uncultured oomycete (HM441908) (aff. Pythium)*
25	*Pythiogeton zeae (HQ665310)*	58	*Uncultured oomycete (HM441654) (aff. Pythium)*	90	*Uncultured oomycete (AY860283) (aff. Pythium)*
26	*Pythiogeton zeae (HQ665310)*	59	*Pythiogeton zeae (HQ665310)*	91	*Uncultured oomycete (AY860253) (aff. Pythiogeton)*
27	*Apodachlya minima (AF235937)*	60	*Pythium sp. (AB468704)*	92	*Apodachlya minima (AF235937)*
28	*Uncultured oomycete (HM442038) (aff. Aphanomyces)*	61	*Aphanomyces cochlioides (HQ665241)*	93	*Pythium conidiophorum (HQ665166)*
29	*Pythiogeton zeae (HQ665310)*	62	*Uncultured oomycete (AY748383) (aff. Pythium)*	94	*Pythiogeton zeae (HQ665310)*
30	*Apodachlya minima (AF235937)*	63	*Uncultured oomycete (HM441337) (aff. Pythium)*	95	*Pythiogeton zeae (HQ665310)*
31	*Pythium ornamentatum (HQ665117)*	64	*Uncultured oomycete (HM442036) (aff. Apodachlya)*	96	*Pythium arrhenomanes (HQ665208)*
32	*Aplanopsis spinosa (AF119589)*	65	*Uncultured oomycete (HM441674) (aff. Pythium)*	97	*Pythiogeton zeae (HQ665310)*
33	*Pythiogeton zeae (HQ665310)*				

*For distributions of OTUs among *americanus* and *australis* populations, see **Figure S2.**
†All affiliations based on BLAST alignments from the GenBank database (Feb 8, 2013). All singletons and doubletons were eliminated from further analysis and are not listed in this table.
