# Supplementary material for: Soil pathogen communities associated with native and non-native Phragmites australis populations in freshwater wetlands
Source: Ecol Evol. 2013 Dec 3;3(16):5254–67. doi: 10.1002/ece3.900 (PMC3892333; doi:10.1002/ece3.900)
Supplement: Supplementary file 7 [file ece30003-5254-SD7.pdf]

Table S4. Reported plant host ranges of oomycete taxa detected in soils from *americanus* and *australis* populations

**Pathogen species**	**Pythium Clade***	**Host range (plant families)**	**Reference**
Aphanomyces astaci	n/a†	None known	n/a
Aphanomyces cochlioides	n/a	Aizoaceae, Amaranthaceae, Caryophyllaceae, Chenopodiaceae, Hydrophyllaceae, Linaceae, Papaveraceae, Portulacaceae, Solanaceae	(Windels 2000)
Aphanomyces piscicida	n/a	None known	n/a
Aplanopsis spinosa	n/a	None known	n/a
Apodachlya brachynema	n/a	None known	n/a
Apodachlya minima	n/a	None known	n/a
Dictyuchus monosporus	n/a	None known	n/a
Halioticida noduliformans	n/a	None known	n/a
Leptolegnia caudata	n/a	None known	n/a
Leptomitus lacteus	n/a	None known	n/a
Phytophthora humicola	n/a	Cucurbitaceae, Pinaceae, Rosaceae	(Ko & Ann 1985; Ginetti *et al.* 2012)
Pythiogeton zeae	n/a	Cucurbitaceae, Poaceae, Solanaceae, Umbelliferae	(Jee, Ho & Cho 2000)
Pythium adhaerens	A	Chenopodiaceae, Cucurbitaceae, Fabaceae, Poaceae	(Sparrow 1932)
Pythium aquatile	B2	Brassicaceae, Solanaceae	(Robertson 1973; Uzuhashi *et al.* 2008)
Pythium arrhenomanes	B1	Poaceae, Cyperaceae, Polygonaceae	(Vanterpool 1940; Dissanayake, Hoy & Griffin 1997; Nechwatal, Wielgoss & Mendgen 2005)
Pythium attrantheridium	F	Fabaceae, Poaceae, Rosaceae, Umbelliferae	(Allain-Boule *et al.* 2004; Broders *et al.* 2007; Reinhart *et al.* 2010)
Pythium conidiophorum	B1	Fabaceae	(Nzungize *et al.* 2011)
Pythium dissotocum	B2	Very broad	(Spencer 2004a)
Pythium heterothallicum	I	Caprifoliaceae, Chenopodiaceae, Fabaceae, Geraniaceae, Poaceae, Rosaceae	(Spencer 2004b)
Pythium monospermum	A	Poaceae, Solanaceae	(Sprague 1950; Robertson 1973)
Pythium myriotylum	B1	Very broad	(Farr & Rossman 2013)
Pythium nagaii	G	Poaceae	(Ito & Tokunaga 1933)

Pythium oopapillum	B2	Very broad	(Bala *et al.* 2010)
Pythium ornamentatum	D	None known	(Spencer 2004a)
Pythium phragmitis	B1	Poaceae	(Nechwatal, Wielgoss & Mendgen 2005)
Pythium porphyrae	A	No plant hosts known	(Spencer 2004c)
Pythium prolatum	H	Ericaceae	(Botha & Crous 1992)
Pythium rhizo-oryzae	B1	None known	(Vanterpool 1940)
Pythium rhizosaccharum	E	None known	(Uzuhashi *et al.* 2008)
Pythium scleroteichum	B1	Convolvulaceae, Euphorbiaceae	(Vanitha & Jacob 1996; Haas *et al.* 2011)
Pythium sylvaticum	F	Very broad	(Spencer 2004d)
Pythium volutum	B1	Poaceae	(Sprague 1950)
Saprolegnia asterophora	n/a	None known	n/a
Saprolegnia unispora	n/a	None known	n/a

* Clade designations according to Levesque et. al. (Levesque & De Cock 2004)
† n/a=not applicable

References

Allain-Boule, N., Levesque, C.A., Martinez, C., Belanger, R.R. & Tweddell, R.J. (2004) Identification of *Pythium* species associated with cavity-spot lesions on carrots in eastern Quebec. *Canadian Journal of Plant Pathology,* **26,** 365-370.

Bala, K., Robideau, G.P., Desaulniers, N., de Cock, A.W.A.M. & Levesque, C.A. (2010) Taxonomy, DNA barcoding and phylogeny of three new species of *Pythium* from Canada. *Persoonia,* **25,** 22-31.

Botha, W.J. & Crous, P.W. (1992) A wilt disease of rhododendron caused by *Pythium prolatum* and *Cylindrocladium scoparum*. *Phytophylactica,* **24,** 75-78.

Broders, K.D., Lipps, P.E., Paul, P.A. & Dorrance, A.E. (2007) Characterization of *Pythium* spp. associated with corn and soybean seed and seedling disease in Ohio. *Plant Disease,* **91,** 727-735.

Dissanayake, N., Hoy, J.W. & Griffin, J.L. (1997) Weed hosts of the sugarcane root rot pathogen, *Pythium arrhenomanes*. *Plant Disease,* **81,** 587-591.

Farr, D.F. & Rossman, A.Y. (2013) Fungal Databases, Systematic Mycology and Microbiology Laboratory, ARS, USDA. Retrieved March 28, 2012, from /fungaldatabases/. Beltsville, MD.

Ginetti, B., Uccello, A., Bracalini, M., Ragazzi, A., Jung, T. & Moricca, S. (2012) Root rot and dieback of *Pinus pinea* caused by *Phytophthora humicola* in Tuscany, central Italy. *Plant Disease,* **96,** 1694-1694.

Haas, S.E., Hooten, M.B., Rizzo, D.M. & Meentemeyer, R.K. (2011) Forest species diversity reduces disease risk in a generalist plant pathogen invasion. *Ecology Letters,* **14,** 1108-1116.

Ito, S. & Tokunaga, Y. (1933) Studies on the rot disease of rice seedlings caused by *Pythium* species. *Journal of the Faculty of Agriculture, Hokkaido University,* **32,** 201-228.

Jee, H.J., Ho, H.H. & Cho, W.D. (2000) *Pythiogeton zeae* sp nov causing root and basal stalk rot of corn in Korea. *Mycologia,* **92,** 522-527.

Ko, W.H. & Ann, P.J. (1985) *Phytophthora humicola*, a new species from soil of a citrus orchard in Taiwan. *Mycologia,* **77,** 631-636.

Levesque, C.A. & De Cock, A.W.A.M. (2004) Molecular phylogeny and taxonomy of the genus *Pythium*. *Mycological Research,* **108,** 1363-1383.

Nechwatal, J., Wielgoss, A. & Mendgen, K. (2005) *Pythium phragmitis* sp nov., a new species close to *P. arrhenomanes* as a pathogen of common reed (*Phragmites australis*). *Mycological Research,* **109,** 1337-1346.

Nzungize, J., Gepts, P., Buruchara, R., Buah, S., Ragama, P., Busogoro, J.P. & Baudoin, J.P. (2011) Pathogenic and molecular characterization of *Pythium* species inducing root rot symptoms of common bean in Rwanda. *African Journal of Microbiology Research,* **5,** 1169-1181.

Reinhart, K.O., Tytgat, T., Van der Putten, W.H. & Clay, K. (2010) Virulence of soil-borne pathogens and invasion by *Prunus serotina*. *New Phytologist,* **186,** 484-495.

Robertson, G.I. (1973) Pathogenicity of *Pythium* spp. to seeds and seedling roots. *New Zealand Journal of Agricultural Research,* **16,** 367-372.
Sparrow, F.K. (1932) Observations on the parasitic ability of certain species of *Pythium*. *Phytopathology,* **22,** 385-390.
Spencer, M.A. (2004a) *Pythium dissotocum*. Descriptions of Fungi and Bacteria. *IMI Descriptions of Fungi and Bacteria,* **Sheet 1613,** 2 p.
Spencer, M.A. (2004b) *Pythium heterothallicum*. Descriptions of Fungi and Bacteria. *IMI Descriptions of Fungi and Bacteria,* **Sheet 1614,** 2 p.
Spencer, M.A. (2004c) *Pythium porphyrae*. Descriptions of Fungi and Bacteria. *IMI Descriptions of Fungi and Bacteria,* **Sheet 1617,** 2 p.
Spencer, M.A. (2004d) *Pythium sylvaticum*. Descriptions of Fungi and Bacteria. *IMI Descriptions of Fungi and Bacteria,* **Sheet 1619,** 2 p.
Sprague, R. (1950) *Diseases of cereals and grasses in North America*. Ronald Press Co., New York.
Uzuhashi, S., Tojo, M., Kobayashi, S., Tokura, K. & Kakishima, M. (2008) First records of *Pythium aquatile* and *P. macrosporum* isolated from soils in Japan. *Mycoscience,* **49,** 276-279.
Vanitha, S. & Jacob, C.K. (1996) Collar rot disease of nursery rubber seedlings, caused by *Pythium scleroteichum* Drechsler. *Indian Journal of Natural Rubber Research,* **9,** 58-58.
Vanterpool, T.C. (1940) Studies on browning root rot of cereals. VI. Further contributions on the effects of various soil amendments on the incidence of the disease in wheat. *Canadian Jour Res Sect C Bot Sci,* **18,** 240-257.
Windels, C. (2000) Aphanomyces root rot on sugar beet. *Plant Health Progress*.
